# Case report: Primary pleural giant extraskeletal Ewing sarcoma in a child

**DOI:** 10.3389/fonc.2023.1137586

**Published:** 2023-03-31

**Authors:** Yang Wu, Chao-Bang Xie, Qiong Huang, Kai-Fei Zhao

**Affiliations:** ^1^ Department of Radiology, The Affiliated Hospital of Zunyi Medical University, Zunyi, China; ^2^ Department of Pathology, The Affiliated Hospital of Zunyi Medical University, Zunyi, China

**Keywords:** extraskeletal Ewing sarcoma, case report, malignancy, intrathoracic tumor, computed tomography

## Abstract

Primary extraskeletal Ewing sarcoma (EES) is a rare small round cell malignancy that accounts for less than 1% of all sarcomas. It is found most commonly in the trunk and lower limbs and very rarely in the pleura and can be easily misdiagnosed in clinical practice. This study presents the case of an 11-year-old boy who presented to our hospital with no apparent cause of left shoulder pain for 6 months. On physical examination, tenderness was noted in the left chest wall and shoulder joint, which had a limited range of motion. Computed tomography (CT) and magnetic resonance imaging (MRI) of the chest revealed an irregular soft tissue mass in the upper left thorax, with a wide base attached to the adjacent pleura and bone destruction of the adjacent left first rib. The patient’s bone scan showed a dense focus of increased radiotracer accumulation in the left first rib. A subsequent CT-guided aspiration biopsy of the left pleural mass with histomorphology and immunohistochemical phenotyping led to a diagnosis of extraskeletal Ewing sarcoma. To inhibit tumor growth, alternating systemic chemotherapy cycles of vincristine, doxorubicin, and cyclophosphamide (VDC) and isocyclophosphamide and etoposide (IE) were administered at 3-week intervals. After completing three VDC and two IE cycles, the child’s condition was well and the pain in the left shoulder joint was relieved. However, a repeat MRI of the chest showed that the mass did not shrink.

## 1 Introduction

Extraskeletal Ewing sarcoma (EES) originating from the pleura is rare and highly malignant, has a poor prognosis, and is difficult to diagnose early. Imaging findings can show the extent of the tumor, local infiltration, and distant metastases and thus guide clinical management.

EES is a rare small round cell malignancy that shares pathology, genetic phenotype, and chromosomal translocation with Ewing sarcoma of the bone and primitive neuroectodermal tumors (1). It comprises less than 1% of all sarcomas, with the most common site of involvement being the trunk and lower limbs. Pleural EES is very rare and easily misdiagnosed clinically. This study describes the case of an 11-year-old boy who presented with left shoulder pain due to a rare giant EES originating from the pleura, with the aim of enhancing our understanding of EES.

## 2 Case description

A previously healthy 11-year-old boy presented to our hospital complaining of left shoulder pain persisting for over 6 months with no obvious precipitating factors such as trauma. He complained of non-radiating paroxysmal dull pain in the left shoulder joint and exertional chest tightness relieved by rest, which was not associated with cough, sputum, or hemoptysis. On physical examination, the chest was symmetrical with no obvious deformity. The neck was supple with the trachea at midline, and the chest expansion was symmetrical. Upon auscultation, hypopnea and wet rales were noted in the left lung field. The left chest wall and shoulder joint area were tender to pressure; the left shoulder joint had a limited range of motion.

### 2.1 Laboratory tests

His glycoantigen-125 level of 61 U/ml (normal reference value: <35 U/ml) was elevated. Alpha-fetoprotein, carcinoembryonic antigen, glycoantigen-199, cytokeratin-19 fragment, ferritin, total prostate-specific antigen, squamous cell carcinoma-associated antigen, and neuron-specific enolase (NSE) levels as well as liver and kidney function test findings and routine blood and coagulation parameters were normal.

### 2.2 Imaging

**Figure 1:**
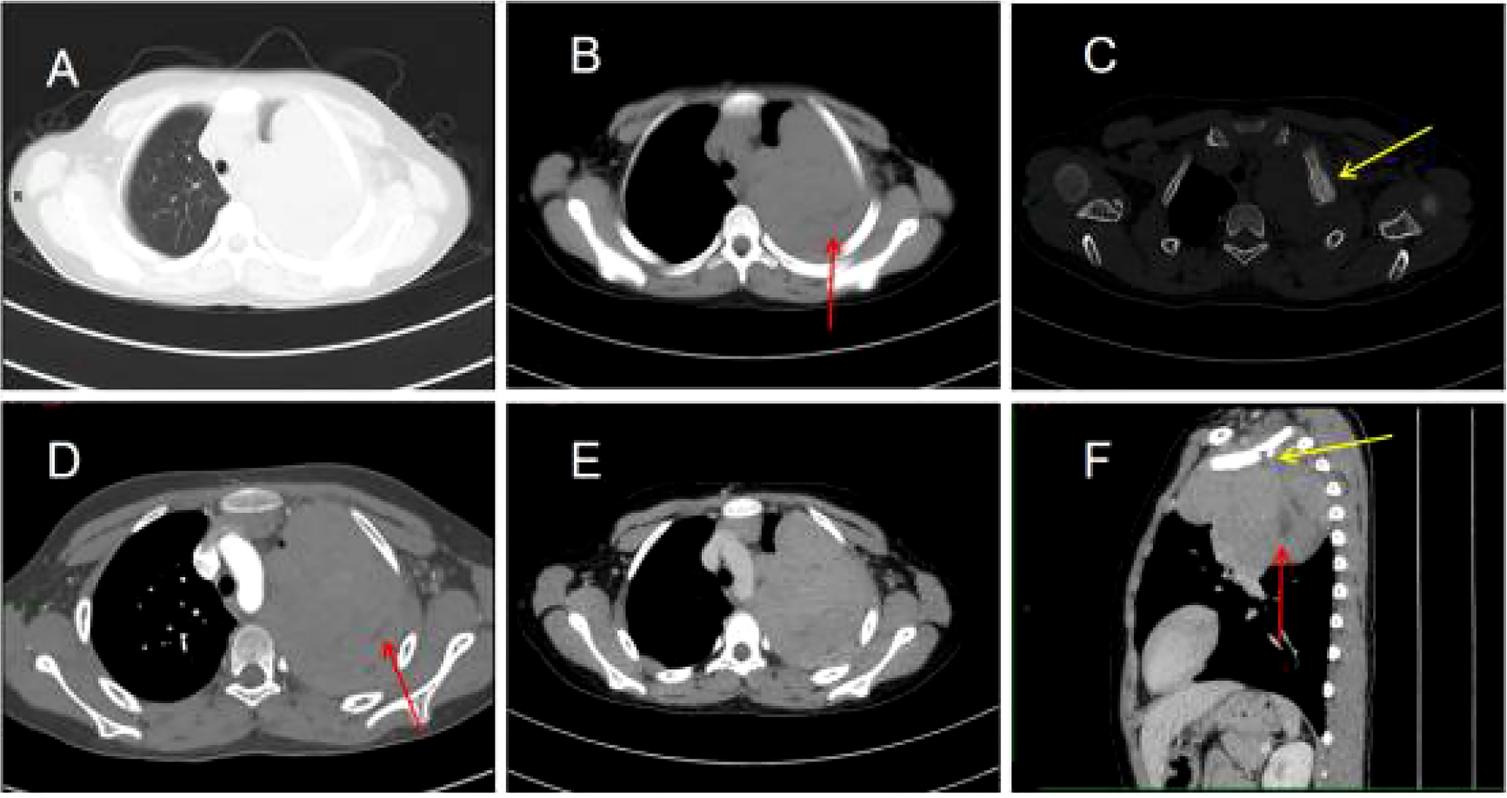
Chest computed tomography (CT) images **(A–F)**. **(A)** Irregular soft tissue density mass in the upper left thorax. **(B)** The mediastinal window shows a lamellar hypodense shadow within the mass (arrow). **(C)** The bone window shows bone destruction of the left first rib and visible periosteal new bone formation (arrow). **(D)** Mild to moderate heterogeneous enhancement of the lesion with non-enhancing hypodense areas within it (arrow). **(E)** Persistent enhancement of the lesion in the venous phase. **(F)** The sagittal section shows bone destruction of the left first rib (yellow arrow) and lamellar hypodense areas within the mass (red arrow).

**Figure 2:**
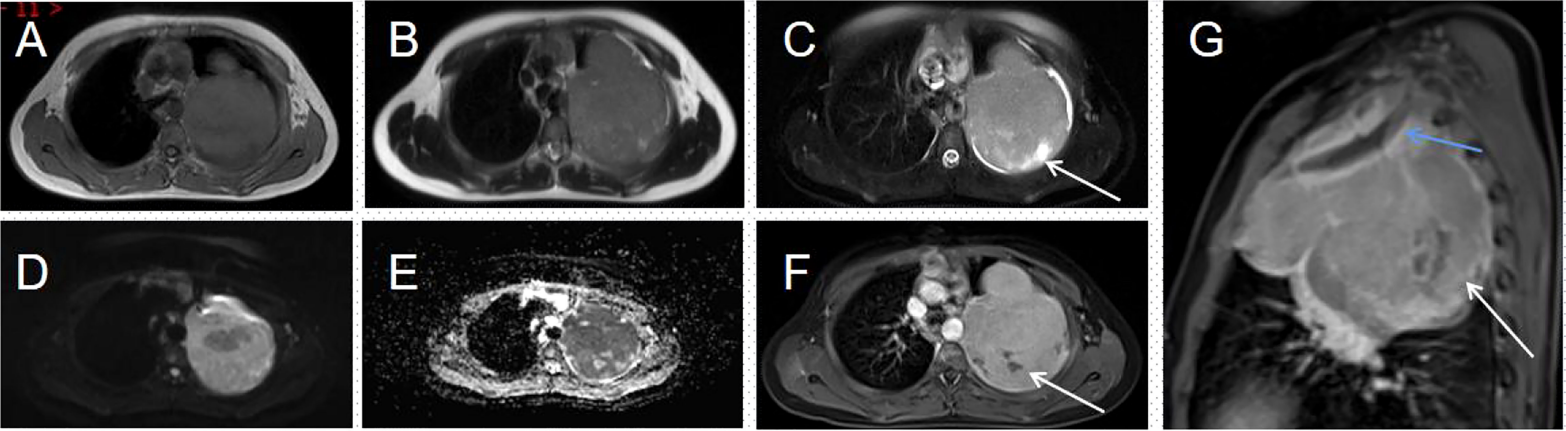
Magnetic resonance imaging (MRI) of the thorax **(A–G)**. **(A, B)** An irregular soft tissue mass is seen in the upper part of the left thorax, showing a mixed signal dominated by iso-T1 and iso-T2 signals. **(C)** T2WI lipid suppression shows a heterogeneous slightly high signal; few long T2 signals are seen within the mass (arrow). **(D)** The solid part of the mass shows a distinct high signal on DWI. **(E)** This corresponds to a low signal on the ADC map. **(F)** The solid part of the lesion is heterogeneously enhanced; no enhancement is seen in the internal liquefied necrotic area (arrow). **(G)** The enhanced sagittal view reveals a bony destruction of the left first rib (blue arrow) and a lamellar area of non-enhancement within the mass (white arrow).

His contrast-enhanced computed tomography (CT) of the chest showed an irregular soft tissue density in the upper left thorax, measuring approximately 85 × 72 × 96 mm, with multiple lamellar hypodense shadows within. Its wide base was connected to the adjacent pleura (**Figure 1**). There was compression of the upper lobe of the left lung; the adjacent left first rib showed bone destruction and visible periosteal new bone formation, with mild–moderate heterogeneous contrast enhancement. Contrast-enhanced magnetic resonance imaging (MRI) showed an irregular soft tissue mass in the upper left thoracic cavity, closely related to the pleura, with mixed signals; predominantly iso-T1 and iso-T2 signals; a slightly high signal with heterogeneous T2 lipid suppression; a few liquefied necrotic areas within the mass, showing slightly long T1 and T2 signals; a significantly high signal in the solid part of the mass on diffusion weight images (DWI), corresponding to a low signal on apparent diffusion coefficient (ADC) maps; and distended bone destruction in the adjacent left first rib (**Figure 2**). The technetium-99m methylene diphosphonate bone scan showed an abnormal focus of intense tracer accumulation in the left first rib. Based on these imaging findings, we considered a left thoracic mass, most likely a malignant tumor of pleural origin, with the invasion of the adjacent left first rib; a pleural sarcoma was our primary differential.

### 2.3 Histopathology

**Figure 3:**
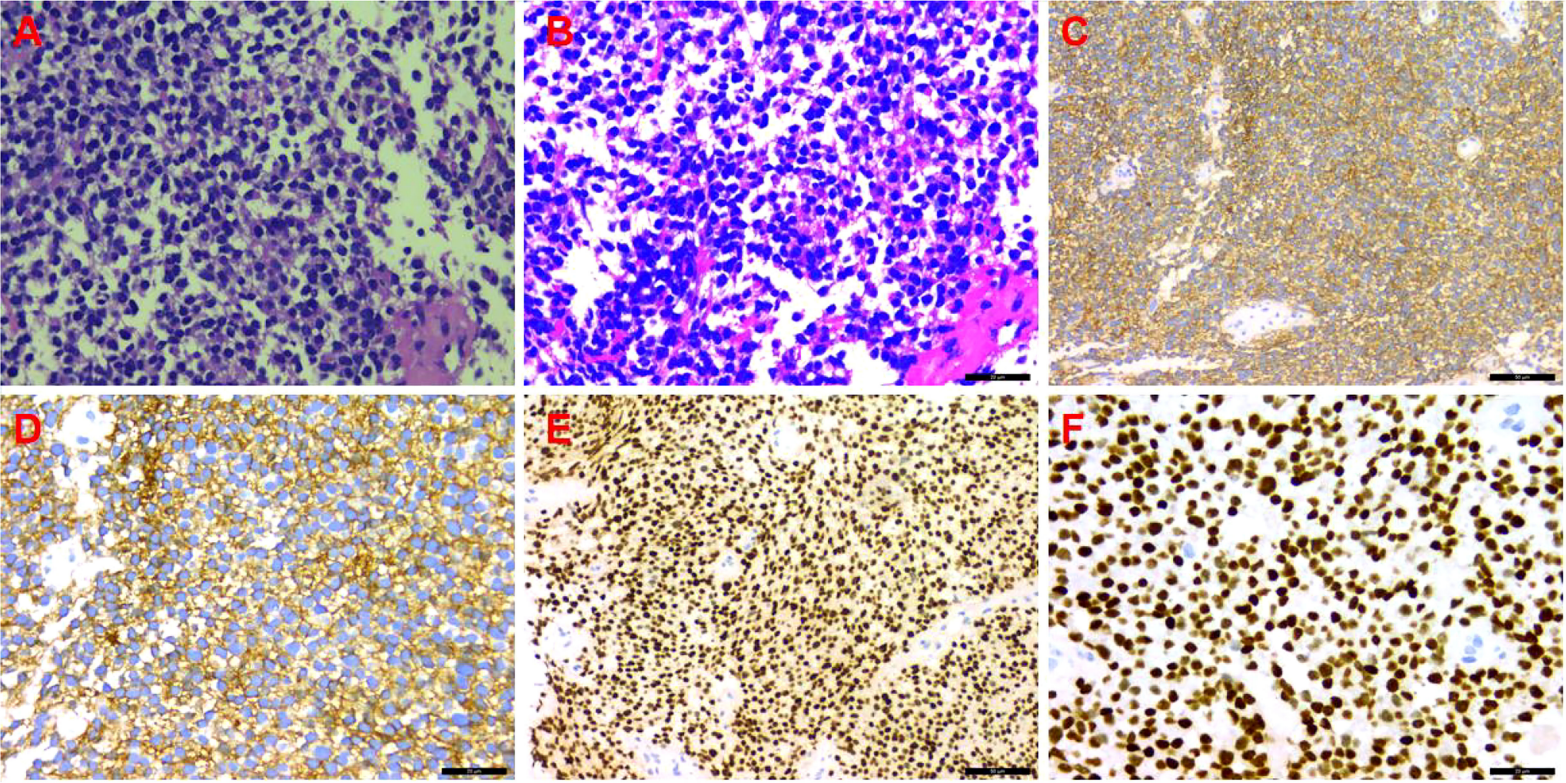
Immunohistochemistry. **(A)** Hematoxylin and eosin (HE) staining (×200); **(B)** HE staining (×400); **(C)** CD99 (++++) (×200); **(D)** CD99 (++++) (×400); **(E)** NKX2.2 (++++) (×200); **(F)** NKX2.2 (++++) (×400).

A CT-guided puncture biopsy of the pleural mass obtained two strips of fish-like tissue. On microscopy, there were small round tumor cells arranged diffusely with some heterogeneous cells. The immunohistochemistry profile was as follows (**Figure 3**): CD99 (++++), vimentin (+), BCL-2 (+), CD117 (+), NKX2.2 (++++), CD56 (−), CK5/6 (−), CD3 (−), CD34 (−), CD20 (−), CD138 (−), CD38 (−), S-100 (−), CK (−), SMA (−), calretinin (−), and Ki-67 (20%). Based on the combined HE morphology and immunohistochemical phenotype, the patient was diagnosed with left pleural EES.

### 2.4 Treatment

**Figure 4:**
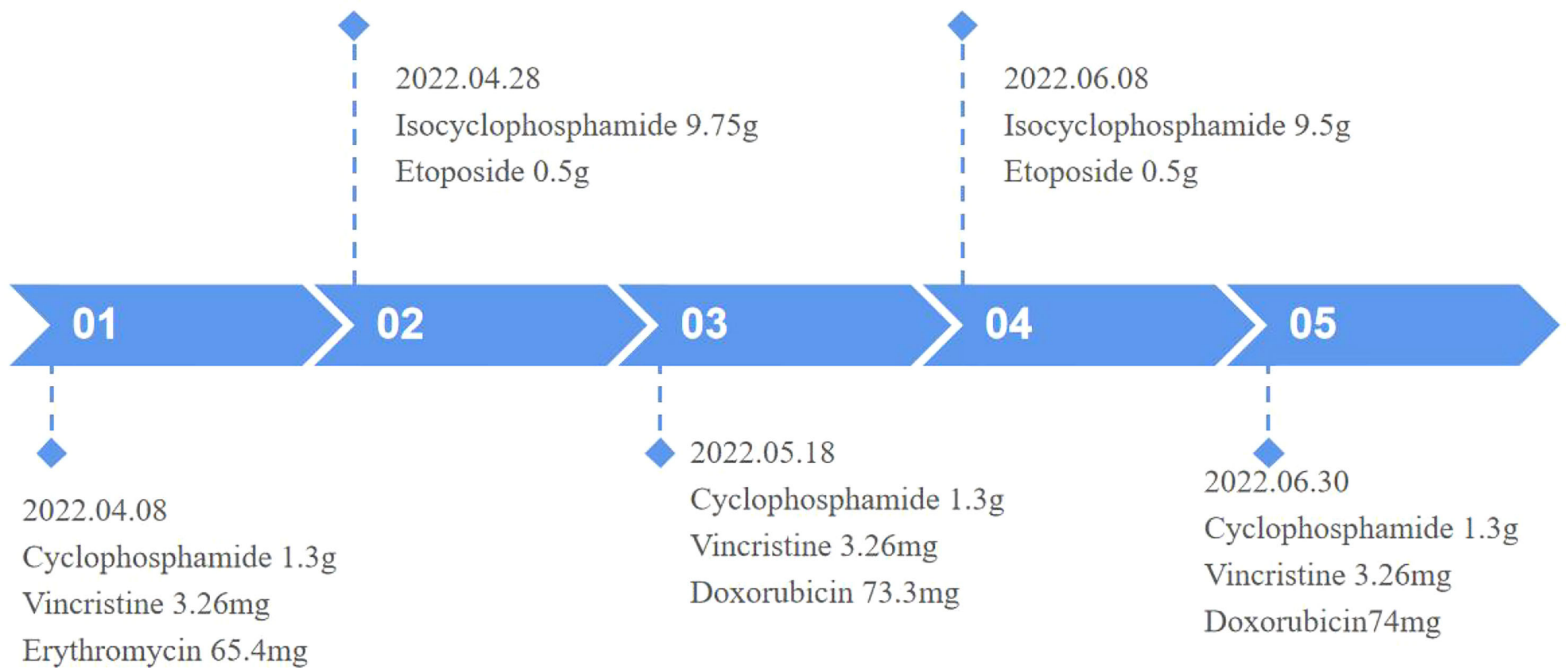
Timeline.

Surgical excision is not considered due to the large size of the mass and its invasion of the adjacent rib, which make surgery difficult. Therefore, to inhibit tumor growth, systemic chemotherapy with alternating programs of vincristine, doxorubicin, and cyclophosphamide (VDC) and isocyclophosphamide and etoposide (IE) was given at 3-week intervals (**Figure 4**). The patient has now completed three VDC and two IE cycles. He was generally well and the pain in the left shoulder joint was relieved; however, a repeat MRI of the chest showed that the mass did not shrink.

## 3 Discussion

EES is a malignant tumor of small round cells in soft tissue, morphologically identical to intraskeletal Ewing sarcoma, with an extremely low incidence of less than 1% of all sarcomas. EES shares pathological, genetic, and phenotypic characteristics and chromosomal translocations with Ewing sarcoma of the bone and primitive neuroectodermal tumors, which are now considered to be of neuroectodermal origin and to belong to the family of peripheral primitive neuroectodermal tumors. Due to its rarity, EES is likely to be misdiagnosed clinically. Primary pleural EES is very rare and occurs in children or adolescents. The clinical symptoms are non-specific and, like other malignant tumors in the chest, present mainly as cough, sputum, and chest pain; thus, they are often initially misdiagnosed as lung cancer. If the mass is large, it may compress or invade the adjacent lung tissue and chest wall, causing symptoms such as chest and back pain, breathlessness, and weakness. In this case, our patient was a young boy who complained of pain in the left shoulder joint, which may be strongly related to the compression and invasion of the huge tumor in the pleural cavity adjacent to the bone destruction. The lung was only compressed and deformed, without obvious cough and sputum symptoms.

On imaging, EES shows up as varisized masses, predominantly larger ones and mostly with smooth margins. CT scans often show a hypo- or isodense mass, which may be associated with cystic degeneration or necrosis resulting in inhomogeneous density within the mass. They are rarely calcified but may stimulate the surrounding bones to form reactive new bone, which appears as pale patchy irregular and slightly dense shadows. After contrast was administered, the mass showed uneven, mild to moderate, progressive enhancement, greater peripherally than at the center and higher in the venous than in the arterial phase; there was no obvious enhancement in the cystic necrotic area. Localization of the mass is possible using the multiplanar reconfiguration technique of multilayer spiral CT. MRI is the imaging method of choice for EES because it shows the location, size, margins of the lesion, and internal signal characteristics of the tumor. T1-weighted imaging (T1WI) is mostly isosignal, with a slightly lower tumor signal than muscle; T2-weighted imaging (T2WI) shows a slightly higher signal; T2 fat suppression shows a significantly higher signal, and sometimes separation is visible; enhancement scans show uniform or heterogeneous strengthening. Due to the high malignancy and rapid growth of the mass, it can invade the adjacent chest wall and destroy the ribs; the mass can also invade the lung tissue and even grow along the interlobular fissures, involving the adjacent lung lobes. EES originating from the pleura often has an envelope and is demarcated from the adjacent lung tissue. It can compress the bronchial wall, causing obstructive pneumonia and atelectasis. The tumor often metastasizes through the lymphatics and rarely hematogenously (2–5). In this case, although the mass was large, most of the margins were well-defined and the enhancement was progressive and uneven. The mass invaded the left first rib and caused periosteal new bone formation. MRI is predominantly iso-T1 and iso-T2 signals, with T2 fat suppression sequences showing high signal and inhomogeneous enhancement on enhancement scans, consistent with the literature.

The presentation of EES on imaging lacks specificity, but CT and MRI are able to localize the tumor, analyze the internal imaging features of the mass, determine its extent, detect invasion of adjacent structures or metastases to distant organs, and evaluate the feasibility of surgery. These are all useful in selecting the optimal treatment options.

The diagnosis of EES is mainly based on histopathology and immunohistochemistry of soft tissue specimens. On light microscopy, the tumor cells are uniform and round, with round or ovoid nuclei; clear nuclear membranes; fine and scattered chromatin; small nucleoli; little cytoplasm; poorly defined, pale eosinophilic red, irregular small vacuoles; and visible nuclear fission phase. The tumor cells are arranged in sheets or indistinct lobules, separated by vascular fibrous tissue. The tumor may sometimes contain chrysalis-like structures, formed by tumor cells arranged around blood vessels, or small nests of intracellular glycogen, located at one end. On electron microscopy, the tumor cells have few intracytoplasmic organelles and abundant glycogen granules, which are still evident on electron microscopy even in PAS-negative specimens. Immunohistochemistry shows that tumor cells express MIC-2, which produces CD99, waveform protein, and FLI-1 as relatively specific indicators for diagnosis, but they do not express S-100 protein, neurofilament protein, UEA-1, etc.; however, some tumors may express NSE (6). It has been reported that NKX2.2 is diffusely positive in EES tissues, significantly higher than in other small round cell tumors, and could serve as a novel marker for the diagnosis and differential diagnosis of EES (7). The expression of translocation chromosome t (11, 22) (q24; q12) and CD99 in EES helps to distinguish it from other small round cell tumors (8). Our patient’s pathological specimens were positive for CD99, waveform protein, and NKX2.2 expression, which is consistent with the diagnosis of EES; unfortunately, no genetic examination was performed.

EES needs to be differentiated from other soft tissue malignancies. Intraosseous Ewing sarcoma has a younger age of onset and is usually located in the long bones of the limbs but also in the flat bones and the paraspinal column, and its imaging manifestations are mainly sieve- and worm-like osteolytic bone destruction with unclear borders and may be accompanied by onion skin-like periosteal reaction, Codman’s triangle, surrounding soft tissue mass formation; osteosclerosis is rare and may invade the adjacent bone. Neuroblastoma also has a relatively younger age of onset and is mainly seen in children younger than 8 years old, especially under 2; its soft tissue masses have a heterogeneous density on CT, with speckled, nodular, or circumscribed calcifications, mixed signals on MRI (T1WI predominantly low signal, T2W predominantly high signal), visible focal necrotic areas, and mild to moderate enhancement, and they may directly invade the chest wall and extend into the adjacent neural foramen. A rhabdomyosarcoma mass is located in the muscle belly, infiltrating and growing along the muscle fibers toward the ends, indistinctly demarcated from the muscle, with liquefaction necrosis in larger masses, rare calcification, and mild or moderate uneven enhancement, slightly higher than the muscle tissue. Malignant lymphoma is seen in older patients with extensive bone destruction, reactive sclerosis, and localized periosteal reaction, surrounded by a distinct soft tissue mass, which is larger than the extent of bone destruction. Pleuropneumoblastoma usually occurs in children under 6 years of age and presents as a large cystic hypodense mass with a thickened wall and areas of liquefied necrosis visible within, with marked inhomogeneous enhancement, and may cause mediastinal displacement (3, 9).

The preferred treatment for EES is a combination of surgical treatment, preoperative neoadjuvant and postoperative chemotherapy, radiotherapy, and targeted drug therapy. The preferred chemotherapy regimen is VDC and IE. If conditions allow, neoadjuvant chemotherapy is recommended before surgery for lesions that are difficult to completely excise, to eliminate microscopic lesions and reduce the size of the local mass, to define the borders of the primary tumor, and to reduce recurrence. Postoperatively, chemotherapy is continued to inhibit the growth of residual tumor tissue (10).

## 4 Conclusion

Pleural EES is very rare. It is highly malignant, has a poor prognosis, and needs to be differentiated from other thoracic malignancies such as neuroblastoma, lymphoma, and embryonal rhabdomyosarcoma, which are difficult to diagnose preoperatively. Confirmation with combined microscopic pathology and immunohistochemistry is recommended. EES grows rapidly. The best treatment in the early stages is surgical excision of the primary followed by local radiotherapy and systemic chemotherapy. In the late stages, local radiotherapy or systemic chemotherapy is the mainstay. There is very little experience on the causes, presentation, and treatment options for pleural EES, leading to a lack of understanding and difficulty in early diagnosis. Imaging can show the extent of the tumor, surrounding infiltration, and distant metastases, providing a reference for treatment.

## Data availability statement

The original contributions presented in the study are included in the article/supplementary material. Further inquiries can be directed to the corresponding author.

## Ethics statement

Written informed consent was obtained from the individual(s)’ and minor(s)’ legal guardian/next of kin, for the publication of any potentially identifiable images or data included in this article.

## Author contributions

YW reviewed the literature and contributed to manuscript drafting. C-BX analyzed and interpreted the patient’s data. K-FZ reviewed and edited the manuscript. QH provided pathological images. All authors contributed to the article and approved the submitted version.

## References

[1] Tefft M Vawter GF Mitus A. Paravertebral "round cell" tumors in children. Radiology (1969) 92:1501–9. doi: 10.1148/92.7.1501 5799839

[2] Javery O Krajewski K Regan K Kis B Giardino A Jagannathan J. A to z of extraskeletal Ewing sarcoma family of tumors in adults: Imaging features of primary disease, metastatic patterns, and treatment responses. AJR. Am J Roentgenol (2011) 197:W1015–1022. doi: 10.2214/AJR.11.6667 22109315

[3] Somarouthu BS Shinagare AB Rosenthal MH Tirumani H Hornick JL Ramaiya NH. Multimodality imaging features, metastatic pattern and clinical outcome in adult extraskeletal Ewing sarcoma: Experience in 26 patients. Br J Radiol (2014) 87:20140123. doi: 10.1259/bjr.20140123 24734938 PMC4075565

[4] Huh J Kim KW Park SJ Kim HJ Lee JS Ha HK. Imaging features of primary tumors and metastatic patterns of the extraskeletal Ewing sarcoma family of tumors in adults: A 17-year experience at a single institution. Korean J Radiol (2015) 16:783–90. doi: 10.3348/kjr.2015.16.4.783 PMC4499542 26175577

[5] Murphey MD Senchak LT Mambalam PK Logie CI Klassen-Fischer MK Kransdorf MJ. From the radiologic pathology archives: ewing sarcoma family of tumors: Radiologic-pathologic correlation. Radiographics (2013) 33:803–31. doi: 10.1148/rg.333135005 23674776

[6] Ashraf MJ Beigomi L Azarpira N Geramizadeh B Khademi B Hakimzadeh A. The small round blue cell tumors of the sinonasal area: Histological and immunohistochemical findings. Iranian Red Crescent Med J (2013) 15:455–61. doi: 10.5812/ircmj.4735 PMC3840830 24349741

[7] Shibuya R Matsuyama A Nakamoto M Shiba E Kasai T Hisaoka M. The combination of CD99 and NKX2.2, a transcriptional target of EWSR1-FLI1, is highly specific for the diagnosis of Ewing sarcoma. Virchows Archiv (2014) 465:599–605. doi: 10.1007/s00428-014-1627-1 25031013

[8] Gurria JP Dasgupta R. Rhabdomyosarcoma and extraosseous Ewing sarcoma. Children (2018) 5(12):165. doi: 10.3390/children5120165 30544742 PMC6306718

[9] Mathew D Prince DN Mahomed N. Extra-skeletal Ewing sarcoma of the chest wall in a child. SA J Radiol (2019) 23:1733. doi: 10.4102/sajr.v23i1.1733 31754538 PMC6837769

[10] DuBois SG Krailo MD Gebhardt MC Donaldson SS Marcus KJ Dormans J. Comparative evaluation of local control strategies in localized Ewing sarcoma of bone: A report from the Children's oncology group. Cancer (2015) 121:467–75. doi: 10.1002/cncr.29065 PMC4305012 25251206

